# Differential Modulation of Hepatic Akt/mTOR Signaling During Acute and Chronic *Toxoplasma gondii* Infection in a Murine Model

**DOI:** 10.3390/cells15100893

**Published:** 2026-05-14

**Authors:** Jianchun Xiao

**Affiliations:** Stanley Division of Developmental Neurovirology, Department of Pediatrics, Johns Hopkins School of Medicine, Baltimore, MD 21287, USA; jxiao4@jhmi.edu; Tel.: +1-410-614-0731; Fax: +1-410-955-3723

**Keywords:** *Toxoplasma*, acute infection, chronic infection, Akt/mTOR, MAG1, cyst burden

## Abstract

**Highlights:**

**What are the main findings?**

- Acute *T. gondii* infection broadly suppresses hepatic Akt/mTOR signaling.
- Chronic infection related to cyst burden activates specific Akt/mTOR nodes.

**What are the implications of the main findings?**

- *T. gondii* has distinct strategies to manipulate host survival based on its life stages.
- The Akt/mTOR pathway may serve as a therapeutic target for the treatment of *T. gondii.*

**Abstract:**

*Toxoplasma gondii* is an obligate intracellular parasite that infects virtually all warm-blooded animals, progressing through acute and chronic stages. The Akt/mTOR signaling axis plays critical roles in cell survival, proliferation, and metabolism, making it a key target for intracellular pathogens. This study investigated how *T. gondii* infection modulates this pathway during both infections. Outbred CD-1 mice were infected intraperitoneally with the virulent GT1 strain of *T. gondii*. Mice for acute studies were sacrificed five days post-infection, while those for chronic studies were treated with sulfadiazine and sacrificed five months post-infection. Phosphoprotein expression of eight Akt/mTOR pathway components was measured in liver tissues using a multiplexed bead-based immunoassay. Acute *T. gondii* infection caused broad suppression of Akt/mTOR signaling, with 6 of 8 markers significantly downregulated, including pS6RP^Ser235/236^, pAKT^S473^, pBAD^Ser136^, pIRS1^S636/639^, pPTEN^Ser380^, and pGSK-3α/β^Ser21/9^. In contrast, chronic infection related to cyst burden selectively activates specific nodes of the pathway, including pBAD^Ser136^, pmTOR^Ser2448^, and pGSK-3α/β^Ser21/9^. Infection induced strong correlations between inter-components, which reflect coherent and coordinated pathway-level reprogramming rather than random perturbation. These findings show that acute and chronic *T. gondii* infections have opposing effects on host Akt/mTOR signaling for their own benefit, which may present new therapeutic targets.

## 1. Introduction

*Toxoplasma gondii* infects approximately one-third of the global human population, making it one of the most prevalent parasitic infections worldwide. The infection develops in two stages: acute and chronic [1]. During an acute infection, rapidly dividing tachyzoites disseminate throughout the host, prompting strong innate and adaptive immune responses to control the multiplication of the parasite. This immune response leads to the conversion of tachyzoites into the slower-growing encysted bradyzoites. Tissue cysts can persist for the lifetime of the host and remain infectious if ingested. Currently, available anti-*T. gondii* drugs are ineffective in clearing the cyst form of the chronic infection [2]. This therapeutic gap largely results from an incomplete understanding of interactions between the host and the parasite during the progression of *T. gondii* infection. Gaining insight into how *T. gondii* infection affects cell survival, metabolism, and proliferation during acute and chronic infection may lead to better approaches. 

*T. gondii* is an obligate intracellular parasite that can infect virtually any nucleated cell in warm-blooded animals. The ability to subvert host cell signaling pathways is central to intracellular parasite survival. *T. gondii* has been shown to hijack host cell biology to survive and replicate by creating a specialized niche, the parasitophorous vacuole (PV), which shields it from lysosomal fusion and degradation [3]. The parasite actively remodels the host cell by secreting effector proteins from specialized organelles (rhoptries and dense granules) directly into the host cytoplasm and nucleus [4]. These effectors manipulate host gene expression, inhibit apoptosis, and subvert host signaling pathways to evade immune responses. Studies on host–parasite interactions have identified numerous signaling pathways altered upon infection.

The phosphatidylinositol 3-kinase (PI3K)/Akt/mammalian target of rapamycin (mTOR) pathway is a master regulator of cell survival, metabolism, and growth [5]. Upon activation by growth factors or insulin receptor signaling, PI3K phosphorylates phosphatidylinositol-4,5-bisphosphate (PIP2) to generate PIP3. This recruits Akt to the plasma membrane, where it is phosphorylated at Thr308 by PDK1 and at Ser473 by the mTOR complex 2 (mTORC2) [6]. Fully activated Akt coordinates diverse downstream responses, including inhibition of apoptosis through phosphorylation of the pro-apoptotic protein BAD at Ser136, which makes BAD dissociate from the Bcl-2/Bcl-X complex and lose the pro-apoptotic function [7]; inhibition of glycogen synthase kinase-3α/β (GSK-3α/β) through phosphorylation at Ser21/9, promoting cell survival and glucose metabolism [8]; and activation of mTOR complex 1 (mTORC1) through multiple mechanisms, leading to phosphorylation of downstream effectors including p70 ribosomal S6 kinase 1 (p70S6K1) at Thr389 and ribosomal protein S6 (S6RP), which drive ribosome biogenesis and protein synthesis. Negative regulation of the pathway is mediated by the tumor suppressor PTEN, which dephosphorylates PIP3 to dampen Akt activation. Additionally, inhibitory phosphorylation of insulin receptor substrate-1 (IRS1) at Ser636/639 acts as a negative feedback mechanism downstream of mTORC1.

Research has suggested that several intracellular pathogens have evolved mechanisms to manipulate the PI3K/Akt/mTOR pathway to their advantage. *Mycobacterium tuberculosis* [9], *Salmonella* [10], and *Listeria* [11] exploit Akt signaling to inhibit apoptosis and promote intracellular survival. PI3K/Akt/mTOR signaling plays a very important role in HPV-induced carcinogenesis by regulating multiple cellular and molecular events [12]. Prior work has demonstrated that *T. gondii* can activate PI3K/Akt/mTOR signaling to facilitate host cell invasion and inhibit apoptosis in infected cells [13,14]. A recent study suggests that the PI3K/Akt/mTOR signaling pathway plays an important role in *T. gondii*-induced mitochondrial dysfunction and the reprogramming of cellular energy metabolism [15]. However, systematic profiling of the complete Akt/mTOR signaling network across both acute and chronic infection stages has not been reported.

The current study systematically profiled the phosphorylation of eight components of the Akt/mTOR pathway in mouse liver tissues, during both acute and chronic *T. gondii* infection, using a multiplexed immunoassay. The hypothesis was that *T. gondii* modulates hepatic Akt/mTOR signaling differently based on its tachyzoite and bradyzoite life stages. The liver is a key metabolic organ and an important site of *T. gondii* infection. During the early stages of infection, tachyzoites disseminate via the bloodstream and infect hepatocytes [16,17]. While significant attention has focused on central nervous system toxoplasmosis, the liver can also harbor bradyzoite cysts during chronic infection [18]. Research has found that the liver is the primary site of tissue pathology [16] in severe toxoplasmosis and can cause liver diseases such as hepatitis [19] and hepatomegaly [20,21]. The present study revealed that tachyzoites rapidly replicate within a metabolically suppressed but still viable host cell, while bradyzoites require a stable, long-lived cellular niche for decades-long persistence.

## 2. Materials and Methods

### 2.1. Ethics Statement

All mouse specimens were collected from prior projects, and no additional animals were sacrificed for the present study. All protocols were approved by the Animal Care and Use Committee at Johns Hopkins University. All experiments conformed to the U.S. National Institutes of Health Guide for the Care and Use of Laboratory Animals.

### 2.2. Acute and Chronic Mouse Models of T. gondii Infection

Six- to eight-week-old female outbred CD-1 mice (ICR-Harlan Sprague) were infected intraperitoneally (i.p.) with 500 *T. gondii* GT1 strain tachyzoites (Type I, virulent). Control mice received vehicle only (PBS). The model employs a type I strain because of its close association with clinical disease and its greater influence on host genes, as demonstrated in our previous research [22,23]. For acute infection, mice were sacrificed at 5 days post infection (dpi), as described previously [24]. For chronic infection, infected mice, including controls, were treated with anti-*T. gondii* chemotherapy (sulfadiazine sodium) in drinking water (400 mg/liter; Sigma) from days 5 to 30 to control tachyzoite proliferation and prevent animal death. Sulfadiazine is a competitive analogue of PABA and inhibits tachyzoite growth, but not encysted bradyzoites. The GT1 strain was maintained by passage in human fibroblast cells (HFF, ATCC SCRC-1041). Mice were sacrificed at five months postinfection (mpi) [25]. Upon sacrifice, liver tissue was immediately harvested, snap-frozen in liquid nitrogen, and stored at −80 °C.

### 2.3. Confirmation of Infection and Cyst Burden Assessment

As described previously [25], *T. gondii* infection in all chronically infected mice was confirmed using a commercial ELISA kit (IB19213, IBL America, Minneapolis, MN, USA) for anti-*T. gondii* IgG antibodies. Cyst burden was quantified using the MAG1 (matrix antigen 1) assay, which measures antibodies to peptide antigens derived from MAG1 [25,26,27]. The MAG1 protein is an antigen found abundantly in the cyst wall and within the cyst matrix. The serological response to the MAG1 peptide antigen has been validated as a proxy marker of chronic *T. gondii* infection and cyst burden [25]. The median MAG1 antibody absorbance value (0.5) was the cutoff to stratify mice into two groups: those with a high cyst burden (OD ≥ 0.5) and those with a low cyst burden (OD < 0.5).

For this study, mice (*n* = 8 per group) were selected based on their antibody profiles. The groups included (i) *T. gondii* unexposed controls (*T. gondii* IgG−/MAG1−) and (ii) *T. gondii* chronically infected mice (*T. gondii* IgG+/MAG1+; IgG OD: IgG = 3.72 ± 0.09; MAG1 OD = 1.291 ± 1.387). The infected mice were further divided into two subgroups: MAG1-high (OD = 2.394 ± 1.153) and MAG1-low (OD = 0.1884 ± 0.1259).

### 2.4. Liver Tissue Homogenization and Protein Extraction

Liver tissue samples (approximately 100 mg) were homogenized on ice in RIPA buffer (Sigma, St. Louis, MO, USA), supplemented with a protease and phosphatase inhibitor cocktail (Thermo Scientific, Waltham, MA, USA). The mixture was sonicated at 4 °C for 5 min, followed by centrifugation at 10,000× *g* for 5 min at 4 °C, and the supernatant was collected. Total protein concentration was determined using a BCA protein assay (Thermo Scientific).

### 2.5. Multiplexed Phosphoprotein Immunoassay

Phosphorylation levels of Akt/mTOR pathway components were measured using a bead-based multiplex immunoassay (Bio-Plex Pro Cell Signaling Akt Panel 8-plex, LQ00006JK0K0RR, Bio-Rad, Hercules, CA, USA) on the Luminex xMAP platform. The panel comprises eight key phosphoproteins located upstream and downstream from Akt and includes: insulin receptor substrate-1 at Ser636/639 (pIRS1^Ser636/Ser639^), phosphatase and tensin homolog at Ser380 (pPTEN^Ser380^), serine/threonine-protein kinase Akt-1 at Ser 473 (pAkt^Ser473^), glycogen synthase kinase-3α/β at Ser21 and Ser9 (pGSK3α/β^Ser21/Ser9^), mTOR at Ser2448 (pmTOR^Ser2448^), p70 S6 kinase at Thr389 (pP70S6K^Thr389^), ribosomal protein S6 kinase beta-1 at Ser235/236 (pS6RP^Ser235/Ser236^), and the Bcl2-associated agonist of cell death at Ser136 (pBAD^Ser136^). All samples were analyzed according to the manufacturer’s protocols, and the median fluorescence intensity (MFI) values were recorded.

### 2.6. Statistical Analysis

All data sets were tested for normality by using the Shapiro-Wilk test. Differences between the two groups were analyzed via Welch’s *t*-test. For multiple groups, ANOVA with Bonferroni’s multiple comparison test was used. Spearman correlation analyses were performed to examine the inter-protein correlation. Multiple testing corrections with a false discovery rate (FDR) of 5% were performed for correlation and infection analysis. Fold change (FC) was calculated as the ratio of the mean MFI of the infection group to the mean MFI of the respective control group. A *p*-value of <0.05 was considered statistically significant; *p* < 0.10 was noted as a trend (†). Data are presented as mean ± standard deviation (SD). All statistical analyses were conducted in Graph-Pad Prism V11.0.0.

## 3. Results

### 3.1. Acute T. gondii Infection Broadly Suppresses Hepatic Akt/mTOR Signaling

To examine the effects of acute *T. gondii* infection on host Akt/mTOR signaling, the phosphorylation levels of eight pathway components in liver tissues were compared between mock-infected and acutely infected mice at 5 dpi.

As shown in Table 1, acute infection resulted in significant suppression of Akt/mTOR signaling across most examined nodes. The most pronounced reductions were observed for p70S6K^Thr389^ (FC = 0.616, *p* = 0.063), pIRS1^Ser636/Ser639^ (FC = 0.636, *p* = 0.0277), pS6RP^Ser235/Ser236^ (FC = 0.630, *p* = 0.0277), and pAKT^Ser473^ (FC = 0.694, *p* = 0.0277), with each showing a 31–38% decrease compared to controls. A moderate decrease was also observed for pBAD^Ser136^ (FC = 0.753, *p* = 0.0277), pGSK-3α/β^Ser21/9^ (FC = 0.783, *p* = 0.0277), and pPTEN^Ser380^ (FC = 0.868, *p* = 0.0435). Notably, pmTOR^Ser2448^ showed a minimal reduction (FC = 0.862) and did not reach statistical significance (*p* = 0.1805). Overall, 6 of 8 markers were significantly downregulated in infected mice, with p70S6K^Thr389^ showing a trend towards reduction.

### 3.2. Chronic T. gondii Infection Related to Cyst Burden Activates Select Hepatic Akt/mTOR Nodes

Chronic *T. gondii* infection activated specific nodes of the Akt/mTOR signaling pathway (Table 2). Compared to mock-infected control, mice that were chronically infected for 5 months showed increased phosphorylation of all 8 components, with significant changes in four: pGSK-3α/β^Ser21/9^ (FC = 1.72, *p* = 0.0016), pmTOR^Ser2448^ (FC = 1.44, *p* = 0.0236), pBAD^Ser136^ (FC = 1.82, *p* = 0.0218), and pAKT^Ser473^ (FC = 1.41, *p* = 0.0218). Additionally, pS6RP^Ser235/236^ showed a trend towards increase (FC = 1.92, *p* = 0.0971).

Chronic infection is defined by the presence of tissue cysts, making cyst burden a critical factor for understanding its effects. I stratified the chronically infected mice into MAG1-high and MAG1-low groups based on MAG1 antibody levels, which serve as a proxy marker of cyst burden [25]. As shown in Table 3, the results indicated that the elevation was particularly pronounced in the MAG1-high mice, with three pathway components showing a significant increase compared to the control group. Specifically, pGSK-3α/β^Ser21/9^ showed a greater than twofold increase (FC = 2.08, *p* < 0.0001), as did pBAD^Ser136^ (FC = 2.29, *p* < 0.05) and pmTOR^Ser2448^ (FC = 1.73, *p* < 0.05). Additionally, p70S6K^Thr389^ (FC = 2.16, ANOVA *p* = 0.097) and pS6RP^Ser235/Ser236^ (FC = 2.57, ANOVA *p* = 0.073) showed a trend towards increase. Moreover, MAG1-high mice also significantly differed from MAG1-low mice in pmTOR^Ser2448^ (FC = 1.51, *p* < 0.05) and pGSK-3α/β^Ser21/9^ (FC = 1.53, *p* < 0.01). No significant difference was found between MAG1-low and mock-infected controls.

None of the eight Akt/mTOR pathway components correlate significantly with MAG1 antibody levels in the chronically infected group (all *p* > 0.05 by Spearman). However, there is a trend indicating a positive correlation between MAG1 antibody levels and pGSK-3α/β^Ser21/9^ (r = 0.46, *p* = 0.0758) or pBAD^Ser136^ (r = 0.43, *p* = 0.096).

### 3.3. Phosphorylation Levels of Akt/mTOR Pathway Components Are Significantly Correlated

**Figure 1:**
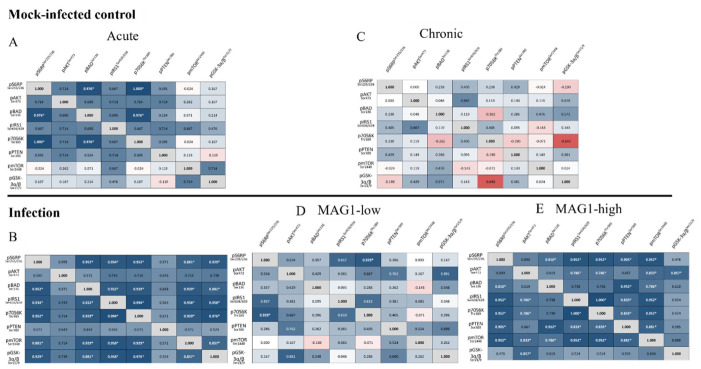
Correlation analyses in mock-infected controls as well as in acutely and chronically infected mice. Spearman correlation analyses followed by multiple testing corrections were performed separately for each group: mock-infected controls during acute infection (**A**), acutely infected mice (**B**), mock-infected controls during chronic infection (**C**), MAG1-low mice (**D**), and MAG1-high mice (**E**) to examine associations among the components measured in the Akt/mTOR pathway. The color scheme of blue, white, and red was utilized to represent the strength and direction of the correlation coefficient. Blue: positive correlation; White: no correlation; Red: negative correlation. bold text with * (*p* < 0.05).

I explored whether activation/inhibition of these Akt/mTOR components was consistent along the pathway. To this aim, correlation analyses were performed among the 8 proteins from both acute and chronic groups, as well as their corresponding controls (Figure 1).

Mice with acute infections or high MAG1 antibody levels show a cohesive phosphoprotein network, with 15 and 17 statistically significant pairwise correlations, respectively. The weakest correlations during acute infection mainly involved pPTEN^Ser380^, whereas in mice with high MAG1 levels, the weakest correlations were primarily with pGSK-3α/β^Ser21/9^. The correlation between p70S6K^Thr389^ and pS6RP^Ser235/Ser236^ is the most robustly coupled link in every infection group (acute, MAG1-low, MAG1-high), confirming tight coupling between the kinase and its substrate during infection. For MAG1-low mice, several pairs show moderate r values but fail the FDR correction, suggesting that pathway coherence is partially maintained, although it is notably weaker. In the mock-infected control for chronic infection, all 28 pairwise correlations are non-significant, while 25 are non-significant in the mock-infected control for acute infection. The correlation between pAKT^Ser473^ and pBAD^Ser136^ remains nonsignificant across all groups, regardless of the infection status.

## 4. Discussion

This study provides the first systematic, comparative analysis of hepatic Akt/mTOR signaling during both acute and chronic *T. gondii* infection in a murine model using the virulent GT1 strain. It revealed that distinct infection stages exert opposing regulatory effects on the host Akt/mTOR pathway: acute infection broadly suppresses activity across most nodes, whereas chronic infection related to cyst burden selectively activates anti-apoptotic and metabolic signaling nodes. These findings are consistent with the distinct functional imperatives of the tachyzoite and bradyzoite life stages [28,29], and support the concept that *T. gondii* has evolved stage-specific strategies to manipulate host cell biology for its own benefit.

### 4.1. Broad Suppression of Akt/mTOR Signaling During Acute Infection

The broad suppression of hepatic Akt/mTOR signaling during acute infection—with 6 of 8 markers significantly reduced at 5 dpi—is consistent with metabolic exploitation and immune evasion during the lytic tachyzoite cycle [30,31].

Upstream of Akt, the reduction in pIRS1^Ser636/639^ appears insufficient to restore downstream Akt signaling, as concurrent activation of pPTEN^Ser380^ occurs. PTEN is a widely known negative regulator of insulin/PI3K signaling [32], so its reduction at Ser380 renders PTEN more enzymatically active, leading to further dephosphorylation of PIP3 and reinforcing suppression of the PI3K/Akt axis. Phosphorylation of IRS1 at Ser636/639 normally results from mTORC1/S6K1-mediated negative feedback on insulin signaling [33]; its reduction most likely reflects the loss of this feedback loop, as supported by reduced p70S6K^Thr389^ and pS6RP^Ser235/236^ expression. This reduction would be expected to enhance IRS1 activity and insulin sensitivity [34]. Pro-inflammatory cytokines (TNF-α, IL-1β, IL-6) activated during acute toxoplasmosis are well-known to interfere with insulin receptor signaling [35].

Downstream of Akt, reduced phosphorylation of BAD^Ser136^ increases pro-apoptotic pressure on the host cell by allowing BAD to interact with the anti-apoptotic proteins Bcl-2 and Bcl-xL [7]. Suppression of Akt signaling may attenuate NF-κB-dependent inflammatory gene expression, since Akt can phosphorylate and activate IκB kinase [36]. Dampening pro-inflammatory responses could thereby delay the onset of effective Th1 immunity. The phosphorylation of GSK3β^Ser9^ or GSK3α^Ser21^ is known to decrease GSK3α/β enzymatic activity [37,38]. The decrease in pGSK-3α/β indicates GSK-3 activation, which reflects a shift away from anabolic glucose storage and pro-survival signaling. The suppression of p70S6K^Thr389^ and pS6RP^Ser235/236^ may redirect biosynthetic resources away from host cell metabolism [39]. This would favor nutrient availability for the rapidly replicating tachyzoite within the parasitophorous vacuole.

Notably, mTOR phosphorylation at Ser2448 was the only node that did not show a significant change during acute infection. As pmTOR^Ser2448^ likely reflects convergent upstream signaling, this implies that mTOR catalytic activity is preserved through Akt-independent mechanisms [40]. This preservation is biologically critical because mTOR is the primary suppressor of autophagy, and its activity prevents autophagic targeting of the parasitophorous vacuole—the host cell’s principal mechanism for intracellular parasite clearance [41,42]. Collectively, this pattern suggests that *T. gondii* has evolved to uncouple mTOR from its upstream Akt activator and converted the hepatocyte into a nutrient-rich but immunologically crippled replication niche.

### 4.2. Selective Activation of Akt/mTOR Nodes During Chronic Infection

In contrast to acute infection, chronic *T. gondii* infection related to cyst burden selectively activates specific nodes of anti-apoptotic (pBAD), metabolic (pmTOR, pGSK-3α/β), and translational (p-70S6K, p-S6RP) pathways. This pattern suggests an overactivated signaling state, supporting the notion that bradyzoites inhibit host cell apoptosis and modify glucose metabolism to create a favorable environment for their survival [28,29]. It is conceivable that MAG1-low mice have insufficient levels of antigens needed to activate these nodes.

The cyst burden-associated activation of pBAD^Ser136^ and pGSK-3α/β^Ser21/Ser9^ carries broad functional implications. Phosphorylation of BAD at Ser136 would enhance anti-apoptotic signaling and maintain host cell viability [43]. Previous studies have found that *T. gondii*-infected cells are broadly resistant to multiple inducers of apoptosis [44]. This resistance may result from Akt and BAD phosphorylation, which, depending on parasite load, inhibit Bax translocation to mitochondria and block apoptosis [14]. Inactivation of GSK-3 may enhance glycolysis and increase glucose availability [45], which is crucial for bradyzoites. GRA18, a dense granule effector protein of *T. gondii*, has been shown to act as an inhibitor of host GSK3, triggering β-catenin accumulation [46]. β-catenin is a key molecule in the Wnt pathway, and its stable expression on Treg cells promotes cell survival [47]. Notably, our previous study found that the phosphorylation levels of the inhibitory forms of GSK-3α/β^Ser21/Ser9^ were decreased in the cerebellum of MAG1-high mice [48], whereas in this study, a more than two-fold increase in the liver was observed. This tissue-specific divergence likely reflects the well-documented pleiotropy of GSK-3 across different cellular contexts.

The significant upregulation of Akt, mTOR, and mTOR-associated targets (p70S6K^Thr389^ and pS6RP^Ser235/236^) in chronically infected mice—particularly in the MAG1-high subgroup—aligns with previous findings of *T. gondii* infection on Akt/mTOR pathway activation [15,49]. Hyperactivation of mTOR has been associated with defects in autophagosome formation, and inhibiting mTOR activity suppresses *T. gondii* replication [50]. A study reports that *T. gondii* inhibits FOXO3a, a transcription factor that regulates the expression of autophagy-related genes, through AKT-dependent phosphorylation [51]. Interestingly, the upstream regulatory machinery of the Akt/mTOR pathway—pIRS1^Ser636/639^ and pPTEN^Ser380^—was not significantly altered during chronic infection, despite robust changes in downstream nodes. The lack of change suggested that upstream PI3K/PTEN regulation may not be the primary driver of chronic-stage Akt activation. Rather, direct activation at or downstream of Akt itself may be responsible and warrants further investigation. Additionally, although cyst burden is a primary factor in the activation of the hepatic Akt/mTOR pathway, there was no significant correlation between phosphorylation levels and MAG1 antibody levels. This suggests that humoral immunity does not directly reflect the intracellular signaling status within the tissue.

### 4.3. Opposing Stage-Specific Strategies

The most striking finding of this study is the diametrically opposed regulatory strategies employed by *T. gondii* between acute and chronic infections. Acute infection broadly suppresses this pathway to redirect host metabolism and dampen immune activation, while chronic infection related to cyst burden selectively activates pro-survival, anti-apoptotic, and anabolic nodes. Similarly, our previous studies also found that *T. gondii* affects host miR-132 differently in acute and chronic infection [24,52]. A study on neonatal mouse astrocytes found that *T. gondii* infection triggers both pro-apoptotic and anti-apoptotic signals [53]. The cells activate apoptosis signals shortly after infection; however, the parasite inhibits programmed cell death for up to 24 h. This delay allows the parasite to replicate, egress, and ultimately cause cellular destruction.

There were highly coordinated changes in the phosphorylation levels of adjacent kinases within the Akt/mTOR pathway in infected groups. This indicates that the integrity of the signaling cascade is maintained in the liver and that the change is not a stochastic phenomenon but a coherent event at the pathway level. While pmTOR^Ser2448^ showed no change in acute infection, it has significant correlations with all other components except pAKT^Ser473^ and pPTEN^Ser380^. Given the correlation between pAKT^Ser473^ and pBAD^Ser136^ is consistently non-significant across all groups, this warrants caution in interpreting BAD as a direct readout of Akt activity. Instead, this implies that other regulatory mechanisms may influence BAD phosphorylation during *T. gondii* infection. Notably, inter-component correlations within control groups differ between the acute and chronic experiments, with acute controls showing more pathway coherence. This difference may reflect age-related changes in hepatic metabolism.

### 4.4. Clinical and Pathological Implications

Previous clinical and epidemiological studies have established associations between *T. gondii* infection—including both acute and chronic toxoplasmosis—and chronic liver diseases, including hepatomegaly, granulomas, hepatitis, cirrhosis, and hepatocellular necrosis in both immunocompetent and immunocompromised patients [54,55,56,57,58]. These findings provide novel molecular mechanistic insight into these diseases, identifying the Akt/mTOR signaling axis as a stage-specific, dynamically regulated target of *T. gondii* infection.

The PI3K/Akt/mTOR pathway is also prominently dysregulated across a wide spectrum of human cancers [59,60]. Given that chronic toxoplasmosis can lead to prolonged overactivation of Akt/mTOR pathways, these findings suggest that long-term *T. gondii* infection could increase oncogenic risk, as noted in other studies [61,62].

This study has several limitations. One limitation is the lack of correlations with conventional liver injury biomarkers, including alanine transaminase (ALT), aspartate aminotransferase (AST), total bilirubin, and alpha-fetoprotein (AFP). Integrating these clinical-biochemical parameters with the phosphoproteomic data reported here would strengthen the translational relevance of these findings. Another limitation is the use of whole-liver homogenate. Although hepatocytes make up nearly 80% of the total cell population [63], the homogenate may also contain contributions from other cell types. Finally, future studies should include total protein normalization, orthogonal validation, and functional assays to strengthen the results.

## 5. Conclusions

In conclusion, this study showed that *T. gondii* exerts stage-specific, opposing regulation of host hepatic Akt/mTOR signaling. These findings reveal the liver as an immunometabolically active site of parasite–host interaction across infection stages. These insights open new avenues for stage-specific therapeutic intervention against toxoplasmosis and provide a molecular framework for understanding the hepatopathological consequences of infection.

## Figures and Tables

**Table 1:** Hepatic Akt/mTOR phosphoprotein levels during acute *T. gondii* infection.

Marker	Control ^a^	Infection ^a^	FC	*p*-Value ^b^	FDR q-Value ^c^	Sig.
pS6RP^Ser235/236^	17,972 ± 5291	11,328 ± 3580	0.630	0.0156	0.0277	*
pAKT^Ser473^	10,347 ± 2556	7179 ± 1473	0.694	0.0131	0.0277	*
pBAD^Ser136^	22,261 ± 2803	16,763 ± 3986	0.753	0.0098	0.0277	*
pIRS1^Ser636/639^	4596 ± 1306	2923 ± 994	0.636	0.0173	0.0277	*
p70S6K^Thr389^	2018 ± 859	1244 ± 475	0.616	0.0557	0.0636	†
pPTEN^Ser380^	10,485 ± 1202	9102 ± 968	0.868	0.0326	0.0435	*
pmTOR^Ser2448^	21,178 ± 2970	18,265 ± 4590	0.862	0.1805	0.1805	ns
pGSK-3α/β^Ser21/9^	10,715 ± 1502	8387 ± 1473	0.783	0.0110	0.0277	*

^a^ Values are mean ± SD (MFI). ^b^ Welch’s *t*-test. ^c^ Multiple testing corrections with a false discovery rate (FDR) set at 5%. † *p* < 0.10 trend; * *p* < 0.05; ns, not significant. *n* = 8 per group. FC, fold change.

**Table 2:** Hepatic Akt/mTOR phosphoprotein levels during chronic *T. gondii* infection.

Marker	Control ^a^	Infection ^a^	FC	*p*-Value ^b^	FDR q- Value ^c^	Sig.
pS6RP^Ser235/236^	3740 ± 1280	7163 ± 6554	1.92	0.0607	0.0971	†
pAKT^Ser473^	2867 ± 380	4036 ± 1475	1.41	0.0077	0.0218	*
pBAD^Ser136^	5565 ± 1082	10,139 ± 5925	1.82	0.0082	0.0218	*
pIRS1^Ser636/639^	1031 ± 60	1454 ± 998	1.41	0.1123	0.1497	ns
p70S6K^Thr389^	580 ± 24	922 ± 865	1.59	0.1347	0.1540	ns
pPTEN^Ser380^	8493 ± 760	8626 ± 2169	1.02	0.8323	0.8323	ns
pmTOR^Ser2448^	4192 ± 550	6042 ± 2522	1.44	0.0118	0.0236	*
pGSK-3α/β^Ser21/9^	3975 ± 765	6840 ± 2307	1.72	0.0002	0.0016	**

^a^ Values are mean ± SD (MFI). ^b^ Welch’s *t*-test. ^c^ Multiple testing corrections with a false discovery rate (FDR) set at 5%. † *p* < 0.10 trend; * *p* < 0.05; ** *p* < 0.01; ns, not significant. *n* = 8 per group. FC, fold change.

**Table 3:** Hepatic Akt/mTOR phosphoprotein levels stratified based on MAG1 group.

Marker	Control ^a^	MAG1-Low ^a^	FC (Low/ctr)	MAG1-High ^a^	FC (High/ctr)	ANOVA p ^b^	Bonferroni Correction
pS6RP^Ser235/236^	3740 ± 1280	4712 ± 2644	1.26	9614 ± 7521	2.57	0.073	—
pAKT^Ser473^	2867 ± 380	3844 ± 599	1.34	4228 ± 1786	1.47	0.105	—
pBAD^Ser136^	5565 ± 1082	7560 ± 3210	1.36	12,719 ± 6290	2.29	0.013	high vs. ctr *
pIRS1^Ser636/639^	1031 ± 60	1103 ± 351	1.07	1805 ± 1141	1.75	0.119	—
p70S6K^Thr389^	580 ± 24	593 ± 110	1.02	1252 ± 1088	2.16	0.097	—
pPTEN^Ser380^	8493 ± 760	7950 ± 893	0.94	9302 ± 2178	1.10	0.338	—
pmTOR^Ser2448^	4192 ± 550	4812 ± 1825	1.15	7272 ± 2585	1.73	0.008	high vs. ctr *, vs. low *
pGSK-3α/β^Ser21/9^	3975 ± 765	5404 ± 1886	1.36	8276 ± 2061	2.08	0.0001	high vs. ctr ***, vs. low **

^a^ Values are mean ± SD (MFI). ^b^ One-way ANOVA followed by Bonferroni’s multiple comparison test among ctr, MAG1-low, and MAG1-high groups. *n* = 8 per group. FC, fold change. * *p* < 0.05; ** *p* < 0.01; *** *p* < 0.001.

## Data Availability

The original contributions presented in this study are included in the article. Further inquiries can be directed to the corresponding author.
